# Considerations and Future Research Directions for E-Cigarette Warnings—Findings from Expert Interviews

**DOI:** 10.3390/ijerph14070781

**Published:** 2017-07-14

**Authors:** Olivia A. Wackowski, David Hammond, Richard J. O’Connor, Andrew A. Strasser, Cristine D. Delnevo

**Affiliations:** 1 Center for Tobacco Studies, Rutgers School of Public Health, Piscataway, NJ 08854, USA; delnevo@sph.rutgers.edu; 2 School of Public Health and Health Systems, University of Waterloo, Waterloo, ON N2L 3G1, Canada; dhammond@uwaterloo.ca; 3 Department of Health Behavior, Roswell Park Cancer Institute, Buffalo, NY 14263, USA; Richard.O’Connor@RoswellPark.org; 4 Department of Psychiatry, Perelman School of Medicine, University of Pennsylvania, Philadelphia, PA 19104, USA; strasse3@mail.med.upenn.edu

**Keywords:** e-cigarettes, warnings, risk communication, health communication, risk perceptions

## Abstract

Tobacco warning labels are important sources of risk information but research historically has been cigarette-centric. This qualitative study aimed to inform future direction and research on warnings for e-cigarettes. Between June and August 2016, we conducted interviews with 10 researchers with expertise in tobacco warning label research. Interviewees were registrants of a 2016 National Cancer Institute grantee meeting on tobacco warnings. Several participants agreed that the Food and Drug Administration’s new nicotine addiction warning for e-cigarettes could be informative but that it might not resonate with young people. Many agreed that more than one warning would be important as e-cigarette science evolves and that research on additional warning themes (e.g., nicotine exposure, harmful constituents) and execution styles (including use of pictorials) was important. Participants were somewhat mixed about the use of reduced-risk messages within e-cigarette warnings, but agreed that research on how to communicate about cigarette/e-cigarette relative risks was needed. Overall, more research is needed on tobacco warnings for non-cigarette products, including on the message content, placement, execution and potential impact on audiences’ product knowledge, risk perceptions and use intentions. This is particularly needed for products such as e-cigarettes which may have harm-reduction potential relative to cigarettes and require unique considerations.

## 1. Introduction

Considerable research has focused on cigarette warning labels, a long standing and highly visible channel for communicating with the public about the risks of smoking [1,2]. Cigarette warning labels may increase consumer knowledge about tobacco risks, discourage initiation and increase quitting behaviors [1,2,3,4]. However, less research exists about warning labels for non-cigarette products, and until recently, no requirements or standards existed in the United States for electronic cigarettes (or “e-cigarettes”) to carry any product warnings, although some brands have done so voluntary [5]. E-cigarettes are novel products that appear to be much less harmful to individual health than tobacco cigarettes [6,7], but are not completely harmless [8]. They pose potential for nicotine addiction and accidental exposure, and may also expose users to some chemicals and carcinogens (although much less so than cigarette smoking) [8,9,10]. Thus, consideration of warnings for e-cigarettes appears relevant. The U.S. Food and Drug Administration (FDA) Center for Tobacco Products deemed e-cigarettes under their regulatory authority in May 2016. The deeming rule requires e-cigarette packaging and advertising to begin carrying one nicotine addiction warning (“WARNING: This product contains nicotine. Nicotine is an addictive chemical.”) within two years [11]. 

Some nascent research suggests that exposure to e-cigarette ads or packaging with nicotine and addiction warnings might increase e-cigarette risk perceptions [12,13] and subsequently reduce willingness to try e-cigarettes [12]. However, two recent studies with differing methodologies each found that e-cigarette addiction warnings might have limited impact. In a focus group study consumers perceived that the FDA’s nicotine addiction warning would not make much impact with smokers or e-cigarette users (because they already know that nicotine is addictive and/or are seeking e-cigarettes for nicotine delivery), but might be important and informative for young people [14]. However, an experimental study found no impact of e-cigarette warnings on young adult non-smokers’ e-cigarette harm perceptions and use intentions [15]. A study heat mapping task conducted within the study suggested the warning did not attract much attention relative to other ad components, and authors suggested that text-only e-cigarette warnings on advertisements might have limited impact on young adult non-smokers’ e-cigarette perceptions. 

Research on the FDA’s nicotine addiction warning as well as other potential e-cigarette warnings is important given that the FDA can propose additional labeling requirements for e-cigarettes in the future. Indeed, the FDA notes it “intends to conduct research and keep abreast of scientific developments” regarding the nicotine warning’s efficacy, information it could use to revise the warning or add any additional warning statements through future rulemaking [11]. However, unique considerations may need to be given to e-cigarette warnings given that e-cigarettes might serve as potential harm-reduction alternatives to tobacco cigarettes among smokers [16]. The final deeming rule also stated that the FDA might consider proposing changes to the warning label requirements in the future depending on emerging research about e-cigarette/cigarette relative risks [11]. Already two tobacco companies have proposed modifying warnings for smokeless tobacco products to indicate they present less risk than cigarettes, though these have not been approved by the FDA [17,18]. In addition, the legal challenges the FDA has faced with regulation over e-cigarettes in general and in trying to implement new high impact graphic warning labels to tobacco cigarettes (criticized as being overly emotional and more extensive than necessary) [19,20] also suggests the need for careful consideration of not only what might be ideal in terms of developing effective e-cigarette warnings, but also legally plausible.

Given the infancy of e-cigarette warnings, this study aimed to inform both researchers and the FDA’s thinking about e-cigarette warnings, relevant unique challenges and potential future research and regulatory directions. As part of our formative work on this topic, we conducted a set of qualitative interviews with tobacco warning label experts to explore their (1) thoughts about the FDA’s nicotine warning; (2) general considerations for e-cigarette warnings; and (3) perceptions about warnings with reduced-risk messages.

## 2. Materials and Methods 

### 2.1. Participants and Recruitment

We conducted semi-structured interviews with a group of ten researchers with expertise in tobacco communication and warning label research, given their potentially more unique familiarity with the purposes and effects of tobacco warnings, best practices for design and effectiveness, and legal and regulatory considerations in the context of warning implementation. Interviewees were registrants of a National Cancer Institute Grantee meeting on tobacco warnings in February 2016 and invited individuals were selected based on our knowledge of their contributions to the field and to include researchers from different academic institutions. Invitations to participate in the current study were sent by email (100% response rate) and the interview questions were sent ahead of time. Interviews were conducted by phone (average 45 min) between June and August 2016 and conducted by the study principal investigator (OW). 

### 2.2. Procedures and Stimulus Materials

Interviews began with discussion about participants’ perceptions of the FDA’s final required e-cigarette nicotine addiction warning (“WARNING: This product contains nicotine. Nicotine is an addictive chemical”). Interviews also asked about perceptions of an example nicotine exposure warning proposed by the FDA in their draft guidance to e-cigarette manufacturers. This warning was suggested as an example warning manufacturers might consider including (and modifying as needed) when submitting e-cigarette product applications to the FDA: “WARNING: Contains nicotine, which can be poisonous. Avoid contact with skin and eyes. Do not drink. Keep out of reach of children and pets. In case of accidental contact, seek medical help [21].” Participants were also asked open-ended questions about their perceptions of other warning concepts and e-cigarette warning issues such as audiences, placement, use of pictorials, and perceived challenges. Participants were also asked their thoughts about using reduced-risk messages in e-cigarette warnings, and about their perceptions of the reduced-risk warning proposed by one snus company (“WARNING: No tobacco product is safe, but this product presents substantially lower risks to health than cigarettes”) in an application to the FDA to obtain modified-risk status for the brand General Snus. 

### 2.3. Analysis

Transcripts were coded with Atlas.ti qualitative software, using codes developed deductively a priori and inductively based on themes, keywords and patterns of responses identified from repeated transcript readings. The principal investigator (OW) coded the interviews and a research assistant reviewed this coded text for consistency and agreement. The coded interviews were then re-reviewed to summarize participants’ responses. Select illustrative participant quotes are presented (in some cases edited for brevity and clarity). This research was approved as an expedited study by the Rutgers Biomedical and Health Sciences Institutional Review Board. 

## 3. Results

### 3.1. Perceptions of the FDA’s Nicotine Addiction Warning

#### 3.1.1. Perceived Strengths

Participants noted that the FDA’s nicotine addiction warning was factual and straightforward and could be helpful in increasing awareness about the presence of nicotine in e-cigarettes, and their potential for addiction.

“…certainly there’s a large number of adolescents who don’t understand that e-cigarettes contain nicotine, so the idea that there be a clear warning is good.”

Some participants also pointed out that the general goals of warnings were to inform the public about potential risks and thus that the audience of an e-cigarette warning could be broad, to include both users and non-users (“…I think it’s important that everybody know, understand, that these products are addictive…”). Others commented that non-tobacco users (people not yet addicted to nicotine) and in particular young people, should likely be priority audiences for e-cigarette warnings.

#### 3.1.2. Potential Limitations

Participants agreed that the nicotine addiction warning likely had several limitations. Some were unsure how readers might interpret it given that addiction was a difficult concept to convey, and one that is colloquially applied to many domains (e.g., sugar, sex, chocolate). In addition, participants noted that the that warning was unlikely to result in any meaningful knowledge gain or behavior change among adult smokers or current e-cigarette users, those most likely to see the warnings, because they are already addicted to nicotine, know about nicotine’s addiction potential, and/or are intentionally seeking out e-cigarettes for nicotine delivery. As such, some speculated that the message might be more relevant for young people or non-smokers but participants expressed concern that the concept of addiction would not resonate with youth. Some referred to the FDA’s Real Cost tobacco prevention campaign, which turned to a “loss of control” theme when formative research found that youth did not relate to addiction. Some also explained that the message could be limited because the writing was passive (“It doesn’t say more directly that nicotine is going to harm my body …”).

One expert commented that the warning was also limited because it is text-only (“…everything that we’ve seen about the text warnings are that they’re useless”) and another that the warning did not acknowledge the issue of abuse liability and that nicotine delivery and addiction might depend on the device used and user. This was noted as representing a challenge for e-cigarette warnings and regulation more broadly, and a topic for future research. 

Despite the comments described above, participants repeatedly acknowledged that there were inherent limitations about what could currently be communicated in e-cigarette warnings given the state of the science (“It’s not the scariest kind of harm but it’s probably one that we know is certainly true…”). Some suggested that even though the warning was limited, it was likely “better than nothing”. Some also noted that its simple, factual, uncontroversial and passive wording (which does not directly claim that e-cigarettes are addictive) is likely intentional to withstand regulatory challenges from the tobacco industry.

### 3.2. Other Warning Concepts

For research and regulation moving forward, participants noted that the goals and audiences of e-cigarette warnings need to be thought through, given that the proposed single message might not resonate with tobacco users nor with non-using young people, and that multiple rotating warnings was good practice for reaching multiple audiences, communicating different health messages and preventing message wearout:

“We need to really clearly think through who are our target audiences and what do we need to do to attract their interest because if they don’t see this message as being relevant to them, then it’s not going to resonate no matter how factually accurate it is. And can this all be done with a single warning message? Very unlikely.”

#### 3.2.1. Other Nicotine Effects

Some thought additional warnings could already be explored. Participants noted that nicotine addiction warnings could be worded in different ways, and that warnings could also describe other negative effects of nicotine, such as on fetal development and on development of the adolescent brain, which might be salient to adolescents. However, one person noted that such a warning might be problematic from a harm-reduction perspective if a similar warning were not also standard for cigarettes. 

With respect to the FDA’s example nicotine exposure warning, participants recognized it as being consistent with poison warnings for other products and generally agreed such a warning seemed reasonable and appropriate for certain e-cigarette products (e.g., e-liquid bottles), and that the inclusion of a Poison Control phone number could also be helpful. However, participants also expressed concern about its long length, which would also likely translate into the use of very small font, both decreasing the likelihood that consumers would actually attend to the warning. Some were unclear how this would be executed in combination with the required addiction warning. While one participant suggested that the warning might work to protect the industry from legal liability, another noted that the industry might challenge it as being too strong or excessive. 

#### 3.2.2. Chemicals and Constituents

Several participants suggested or agreed that tobacco control professionals could consider crafting and testing warnings about the presence of constituents/chemicals in e-cigarettes other than nicotine. In addition, such messages could indicate what other substance or products those chemicals were found in or known health effects of those chemicals. Such an approach could be a relevant way to communicate about the potential risk of e-cigarettes in the absence of data about the long-term effects of e-cigarette use. 

However, participants again agreed that such messages would have to be consistent with the science on e-cigarette constituents and could be challenging given the variability of product types. One person noted that such messaging might be more feasible in the future as the e-cigarette market consolidates under FDA regulation. Participants also warned about over-stating the risks of e-cigarette chemicals and e-cigarette risks in general in the context of their potential use for harm-reduction by current smokers.

#### 3.2.3. Explosion Risk

Participants generally did not think that a message about the risk of explosions and/or burns from e-cigarettes would be appropriate for a mandated warning given that they were relatively rare, might be conditional on specific products/devices or consumers’ specific product use/charging behaviors and that ultimately, for these various reasons, would be difficult to defend in court. One expert noted these risks should be controlled by product regulation rather than a warning. 

#### 3.2.4. Cessation-Related

Similarly, most participants agreed that a message warning that e-cigarettes are “not approved for smoking cessation” would not work as a warning because it could be true for some products and e-cigarettes might be effective for smoking cessation for some users. It was also noted that it was more of a disclaimer messages that some manufacturers might use, rather than a warning that regulators should require on all products. Some participants, however, stated that this message is important to distribute in that it could inform consumers that we do not yet know if these products help people quit smoking. 

### 3.3. Potential Advantages of Pictorial and Colored Warnings

Participants generally agreed that use of pictorials in tobacco warnings were important for increasing their attention, salience, and memorability and therefore should also be considered for e-cigarette warnings:

“we’re helping people to at least pay attention to the messages, to remember them, hopefully remember them over time. And really it’s imagery that helps people do that. So I would say absolutely use images.”

Participants suggested that symbols associated with poison and danger could be tested for the nicotine exposure warning (e.g., skull and crossbones) or other universally understood hazard or warning symbols. Some commented that addiction was more challenging to depict (and might be difficult to defend in legal challenges) but that various symbolic associations could be explored (e.g., images of chains, handcuffs, needles). However, one person noted that pictorial warnings should not be used for e-cigarettes before being implemented for tobacco cigarettes in the United States. Another stated that if pictorials are used for e-cigarettes, that they should be specified by the FDA and standardized for all e-cigarette products, not left up to individual manufacturers to decide on, as seemed to be suggested in the draft guidance for the nicotine exposure warning. 

Some participants also supported the idea of researching whether colored text warnings (such as warnings with a yellow, orange or red background or colored font) might be useful in terms of attracting visual attention.

“…we do a lot of things in black and white, but you know safety orange conveys a message, other bright colors convey warning. People associate those colors with danger and warning. And so there may be some interesting variations that could be done that would make it much more visible and would stand out….”

However, it was also acknowledged that picking a warning color that would contrast the packaging might be difficult given the lack of standardized packaging, and that the tobacco industry would likely try to “redesign their packaging in a way to minimize, hide, and undermine any warning that we put on them”.

### 3.4. Warning Placement and Exposure

Participants generally agreed that e-cigarette warnings were likely to be a less efficient channel for communicating with the public about product risks compared to cigarette package warnings because they provided fewer opportunities for exposure. For example, a user might view an e-cigarette warning on the packaging of a vaporizer device when first opening it, but then no longer carry the device in that packaging. Similarly, users might view e-cigarette warnings when interacting with bottles of e-liquids to refill their devices, but not regularly carry these bottles/vials with them like cigarette packs. However, one participant noted that this did not mean such warnings would not be useful, and perhaps instead underscored the importance of making e-cigarette warnings as salient as possible:

“You know [this] speaks to the importance of making sure that if the warnings are on the packaging, the packaging that will be disposed of … that you make sure that they are as noticeable and salient as possible because you’re just not going to get the same level of exposure [as with cigarette warnings].”

The small size of e-liquid containers was also noted as a challenge for placing warnings large enough to be readable. Some participants suggested that research should explore the use of packaging inserts for e-cigarette risk communication. 

### 3.5. Reduced-Risk Warnings

Participants were asked about their perceptions of the reduced-risk warning proposed by Swedish Match for its General Snus products (“WARNING: No tobacco product is safe, but this product presents substantially lower risks to health than cigarettes”), as well as the idea of having reduced-risk warnings for e-cigarettes. Some noted that the General Snus message was true and reasonable and were supportive or at least open to the concept of reduced-risk warnings for snus and e-cigarettes given their harm-reduction potential. However, participants also expressed uncertainty about how people would respond to such a message and concerns that some, particularly young people, might misinterpret it as meaning the product is safe and as a product endorsement. Similarly, participants were not sure that such a message should be included within a product warning, versus elsewhere on advertising:

“I think simply using the warning label or that area for endorsing the product, that’s not the right purpose for the warning. The ‘safer product’ could be done with the communications from the company, the advertisements ….”

Some also commented on specific wording aspects of the General Snus warning, noting that “substantially” was a high literacy word (which could likely be replaced with something simpler such as “much”). One person suggested that the “messaging probably needs to consider the issue of dual-use or poly-use” (e.g., “this product could present substantially lower risks to health if you were to use it exclusively compared to using cigarettes exclusively”). Another commented that the statement should be more specific/active (“it says ‘no tobacco product is safe’. I think it should say ‘this product is not safe’) and should use the word “harm” rather than “risk”:

“Because people don’t know risk. I mean risk is probabilistic … you know, risk is like “oh it might happen, it might not”. Harm, that’s an outcome. Risk is not an outcome.”

Another participant who was open to the idea of reduced-risk messages for snus and e-cigarettes similarly suggested that such messages should probably lean toward and emphasize the warning part of the message, rather than the reduced-harm part of the message:

“…I think the emphasis needs to fall on you know ‘these products may be less harmful, but they’re harmful’. I mean I think the emphasis will always fall on communicating a level of harm as the primary message and the secondary one giving some relative-risk compared to cigarettes. So you know I think the Swedish Match warning doesn’t do that.”

Overall, participants noted that the impact of such messages for products such as smokeless tobacco and e-cigarettes, presented in different wording and executions styles (as even subtle differences could be perceived differently), was a very important area for research, and one which was currently very limited:

“I think that would be something really valuable for the FDA to know about … and necessary for us to kind of build the evidence base for what might be a sensible approach. And if not for FDA, which might have their hands tied, for other governments throughout the world where there might be greater flexibility.”

In the meantime, one person explained that even without an explicit reduced-risk message for e-cigarettes, lower risk could be implied in other ways:

“In most other countries, cigarettes have bigger warnings and they have pictures, whereas smokeless products do not. And so irrespective of any relative-risk content, it is true that consumers perceive the size of warnings as an indicator of the severity of risks. So I think there are ways of doing that through the design of the warning.”

## 4. Discussion

This study points to challenges and avenues for future research about e-cigarette warnings. E-cigarettes pose the potential for nicotine addiction and accidental exposure, and may also expose users to some chemicals and carcinogens [8,9,10]. However, the science on potential e-cigarette health effects is nascent and research thus far suggests that they appear to be much less harmful to individual health than tobacco cigarettes [6,7]. Furthermore, the e-cigarette market is large and nicotine delivery and exposure to potentially harmful constituents can vary greatly by product type (e.g., size, voltage) and user behavior [9]. 

Given the state of the science, the FDA’s required nicotine addiction warning appears to be an appropriate starting point for informing consumers about the potential risks of e-cigarettes. However, experts in this study generally agreed that the FDA and researchers should consider and study the use of additional warnings moving forward as e-cigarette science progresses. Experts also raised concerns that the starting nicotine addiction warning might not resonate with young people, who are intended to be a priority audience for it. Future research should explore variations to the execution of this warning (e.g., in terms or wording or use of graphics) that might enhance its noticeability and salience with young people in particular. Some suggested that warning about the potential effects of nicotine on the adolescent brain (a concern discussed in the most recent Surgeon General’s report) [8] might be a meaningful theme to young people and worth researching in the future. 

In addition, this study suggests that future research should consider exploring the use of pictorials and/or colored text warnings for e-cigarettes to maximize their attention, especially given that they likely pose fewer exposure opportunities compared to tobacco cigarettes. Indeed, a previous study of e-cigarette warnings found that non-smokers did not appear to attend to text-only addiction warnings on e-cigarette ads [15]. While this might be a challenge given legal difficulties in implementing pictorial warnings for tobacco cigarettes, the previous rulings against graphic cigarette warnings related to the specific images used and not the government’s use of graphic warnings in principle [22]. As such, potential pictorials for e-cigarette warnings could be planned appropriately, perhaps making use of symbols associated with addiction (e.g., chains, brain) or caution (e.g., yellow triangle) [23] rather than graphic health effects or negative emotions (e.g., person crying) proposed for tobacco cigarettes that might be criticized as “overly emotional” or “more extensive than necessary” [19,20]. Alternatively, if young people are the primary audience for e-cigarette warnings, pictorials could potentially depict images of young people to both attract attention and communicate that the message is aimed at them (unless formative research finds this unintentionally increases young peoples’ product interest). Future research could explore these ideas through experimental and eye-tracking studies. 

Consistent with previous consumer research [16], we also found that although many of the experts were open to communication about the reduced-risks of e-cigarettes relative to cigarettes, they were somewhat mixed and unsure about the inclusion of reduced-risk messages within warning labels, as has been proposed by two tobacco companies to date [17,18]. Reduced-risk messages presented within warnings may potentially water down the warning, be confusing, and no longer act as a product “warning” per se [16]. In contrast, it might be argued that providing reduced-risk information in close proximity to e-cigarette risk information puts the risk information in appropriate context. It is also possible that reduced-risk information might be perceived as more believable when presented within a warning than as an industry claim elsewhere on the product packaging or advertising. Overall, research is needed to better understand how to communicate tobacco product relative risks, where these communications should be placed, and their potential impact on consumer product perceptions and use intentions, including among tobacco users and non-users. 

Another direction for future research could be the use of inserts with e-cigarette packaging, as suggested by study participants. Indeed, since 2000, Canada has supplemented warnings on tobacco packages with information on inserts inside the packaging [24], and inserts might be particularly useful for e-cigarettes to address information that might not be appropriate or practical for small warning labels such as how to safely charge and store the product and its batteries, what to do in case of accidental nicotine exposure, nuanced information about the potential varying degree of risks/exposures based on product type and caveats of any included reduced-risk messages (e.g., conditional on complete product switching, not dual use).

Finally, although consideration of warnings for e-cigarettes is warranted given the growth in their use [8,9], and interest in their risks and safety [25,26], the fact remains that e-cigarettes are likely much less harmful to individual health than tobacco cigarettes. For the United States, this means that efforts to strengthen the current small text-only warnings on tobacco cigarettes must continue, to follow over 100 countries around the world with large pictorial cigarette warnings [27]. Furthermore, text warning requirements on smokeless tobacco products were strengthened in 2010 (i.e., increasing label size to 30% of packaging front and backs), creating an unusual situation in the US where smokeless tobacco products have more prominent warnings than conventional cigarettes (which remain unchanged since 1984) [22]. If changes are not implemented soon, then by 2018 e-cigarette packaging may also carry more visually prominent warnings than cigarettes. Some e-cigarette brands with voluntary labels, such as MarkTen, already do [5,14]. This discrepancy may contribute to increasing perceptions that e-cigarettes are as harmful as tobacco cigarettes [28].

## 5. Conclusions

Overall, this study builds on previous research to provide insight into considerations for developing and researching e-cigarette warnings. In developing e-cigarette warnings, we should draw from what is known about warning label effectiveness but also tread carefully given their harm reduction potential. Ideally, such warnings should increase awareness about potential e-cigarette risks and discourage e-cigarette use among never smokers while not discouraging use among current and former smokers interested in e-cigarettes for smoking cessation or maintaining abstinence [14]. Research on ways to communicate about e-cigarettes risks relative to cigarettes is also very important moving forward.
